# NAD^+^ boosting increases atherosclerotic plaques and inflammation in *Apoe* knockout mice

**DOI:** 10.1016/j.atherosclerosis.2025.119188

**Published:** 2025-04-03

**Authors:** Yu-Jen Wang, Daniel S. Gaul, Era Gorica, Jürgen Pahla, Zeneng Wang, Shafeeq A. Mohammed, Tina Dahlby, Elisa Dietrich, Elena Osto, Karim Gariani, Sarah Costantino, Stephan Winnik, Sokrates Stein, Stanley L. Hazen, Frank Ruschitzka, Johan Auwerx, Christian M. Matter

**Affiliations:** a Center for Translational and Experimental Cardiology (CTEC), Department of Cardiology, University Hospital Zurich and University of Zurich, Zurich, Switzerland; b Department of Cardiology, University Heart Center Zurich, University Hospital Zurich, Zurich, Switzerland; c Department of Cardiovascular & Metabolic Sciences, Lerner Research Institute, Cleveland Clinic, Cleveland, OH, USA; d Institute of Food, Nutrition and Health, ETH Zurich, Zurich, Switzerland; e Institute of Clinical Chemistry, University Hospital Zurich and University of Zurich, Zurich, Switzerland; f Division of Physiology and Pathophysiology, Otto Loewi Research Center for Vascular Biology, Immunology and Inflammation. Medical University of Graz, Graz, Austria; g Service of Endocrinology, Diabetes, Nutrition, and Therapeutic Education, Faculty of Medicine, Geneva University Hospitals, Geneva, Switzerland; h Department of Cardiovascular Medicine, Heart, Vascular and Thoracic Institute, Cleveland Clinic, Cleveland, OH, USA; i Laboratory of Integrative Systems Physiology, École Polytechnique Fédérale de Lausanne, Lausanne, Switzerland

**Keywords:** Nicotinamide riboside (NR), Nicotinamide adenine dinucleotide (NAD^+^), Atherosclerosis, Liver, Macrophage, CD38

## Abstract

**Background and aims:**

NAD^+^ (nicotinamide adenine dinucleotide) is a cosubstrate of the sirtuins (SIRT) that are activated upon caloric restriction. Supplementing NAD^+^ precursors such as nicotinamide riboside (NR) has been reported to extend life span and combat metabolic syndrome through *pan*-sirtuin activation in mice. Notably, sirtuins compete with poly (ADP-ribose) polymerase (PARP)1 and CD38 for NAD^+^. Supplementing NAD^+^ precursors did not improve cardiovascular outcome in the AIM-HIGH trial. Recently, the terminal NAD^+^ metabolite 4PY (*N*^1^-methyl-4-pyridone-3-carboxamide) was reported to increase inflammation and to be associated with cardiovascular risk. We aimed to investigate whether NR provides atheroprotection.

**Methods:**

8-week-old male apolipoprotein E (*Apoe*) knockout mice were fed for 12 weeks a high-cholesterol diet supplemented with three NR doses: NR−, NR+, and NR++. RAW264.7 mouse macrophages and bone marrow macrophages were stimulated with oxLDL and NR.

**Results:**

NR++ enhanced plaque lesions in aortic sinus sections and increased plasma levels of TNFα, IL-6, and LDL-cholesterol. Liver and plasma NAD^+^ concentrations remained unchanged, but the downstream metabolite 4PY increased. In liver lysates, SIRT1 and lipoprotein receptors were decreased and CD38 increased in NR++; cleaved PARP1 and total PARylation decreased upon NR supplementation. In oxLDL-treated macrophages, high NR levels increased CD38 and CD86 expression.

**Conclusions:**

High-dose NR supplementation in mice did not decrease but increase both aortic plaque lesions and systemic inflammation. These effects may be mediated by increased CD38 expression in macrophages, with NAD^+^ metabolism shifted from sirtuins towards CD38 and PARP1 pathways. Caution should be applied with presumed NAD^+^ boosters in patients with atherosclerosis.

## 1. Introduction

NAD^+^ (nicotinamide adenine dinucleotide) is the electron carrier of numerous oxidation reduction reactions, whereby the ratio to its reduced form NADH reflects the cellular energy state. NAD^+^ is also a cosubstrate of a variety of enzymes, thus linking enzyme function and cellular energy metabolism. Sirtuins (SIRT) are one group of these NAD^+^-consuming enzymes, functioning as NAD^+^-dependent deacetylases. Sirtuins are activated upon caloric restriction and exert protective effects on atherothrombosis through immunometabolic pathways [1]. Other NAD^+^-consuming enzymes include PARP1 (poly-[ADP-ribose] polymerase-1) and CD38. PARP1 is involved in DNA repair upon metabolic and oxidative stress and CD38 is expressed ubiquitously in immune cells and consumes NAD^+^ extracellularly [2].

Seven sirtuins (SIRT1–7) have been identified with different subcellular expression patterns. Both direct activation of individual sirtuins or genetic overexpression have shown beneficial effects [1,3,4]. Yet, it is challenging to design SIRT-activating compounds with specificity and efficacy for clinical application [5]. Increasing the cosubstrate NAD ^+^ pool to *pan-*activate all sirtuins have been proposed and termed as NAD^+^ boosting. NAD^+^ boosting has been reported to extend health span and combat immunometabolic diseases [6,7]. Nicotinamide riboside (NR) is a naturally occurring precursor of NAD^+^ [8]. NR can be phosphorylated and transported into cells, where it is synthesized into NAD^+^ and fuels the cellular NAD^+^ pool [9]. NR supplementation has been shown to combat metabolic syndromes in preclinical studies including mouse models of obesity [10] and liver steatosis [11].

However, in clinical trials, NAD^+^ boosting falls behind expectations. Niacin, another precursor of NAD^+^, did not reduce residual risk of cardiovascular diseases in AIM-HIGH [12] trial albeit raising high-density lipoprotein (HDL) and lowering low-density lipoprotein (LDL) levels in combination with statin treatment. [13] Moreover, recent clinical and experimental studies showed that increased levels of the NAD^+^ terminal metabolite 4PY (*N*^1^-methyl-4-pyridone-3-carboxamide) both fostered vascular inflammation *in vivo* and is associated with heightened risk for major adverse cardiovascular events [14]. While some smaller trials with niacin in the setting of hyperlipidemia have shown moderate reduction in pro-inflammatory cytokine levels, but no other cardiometabolic endpoints were achieved [15,16].

Taken together, the current reports about NAD^+^ boosting in the context of atherogenesis remain controversial. Thus, we hypothesized that NR supplementation results in atheroprotection. We tested this hypothesis by investigating a dose-response of NR supplementation on plaque formation and corresponding immunometabolic pathways in atherosclerotic mice.

## 2. Materials and methods

### 2.1. Animals and diets

Mice were housed in cages with free access to experiment diets and water in a temperature-controlled facility with a 12-h light/dark cycle. All experiments and animal care procedures were approved by the local veterinary authorities and carried out in accordance with institutional guidelines. (License number: 239/2013 and ZH023/17) C57BL/6J male apolipoprotein E knockout (*Apoe*^−/−^) mice were used in this study. *Apoe*^−*/*−^ mice underwent 12 weeks of high-cholesterol diet (1.25 wt%) treatment and littermates were randomly assigned to three different groups, which were supplemented with 0, 1.2, or 2.4 g/kg diet of nicotinamide riboside (NR, Niagen^®^, ChromaDex), for NR−, NR+, NR++ group respectively (Research Diets). Body weight was monitored every two weeks. After 15 h of fasting (18:00 p.m.−09:00 a.m.), the mice were euthanized, and blood was collected in EDTA (Ethylenediaminetetraacetic acid) tubes and centrifuged to obtain plasma. Mice were then perfused with cold PBS (phosphate-buffered saline), and other organs were harvested and snap-frozen in liquid nitrogen. Frozen plasma and tissue/organs were kept at −80 °C until further analyses.

### 2.2. Cell culture and treatment

Tibiae and femora were isolated from wild-type mice. The bones were centrifuged to spin out the bone marrow cells. RAW264.7 cells (Sigma-Aldrich) and isolated bone marrow cells were cultured with high-glucose DMEM (Sigma, D5796), supplemented with 10% fetal bovine serum (FBS, Gibco) and Penicillin/Streptomycin (100 U and 0.1 mg/mL, Sigma, P0781). Cells were incubated at 37 °C, 5% CO_2_, in a humified incubator. For treatment, oxLDL (oxidized low-density lipoprotein, KALEN Biomedical, 770252) and/or NR (Sigma-Aldrich, SMB00907) were dissolved in starvation medium (2% FBS in DMEM for RAW264.7 cells) or complete medium (for bone marrow cells) and cells were treated for 24 h before analysis.

### 2.3. Atherosclerosis quantification

**Fig. 1.**
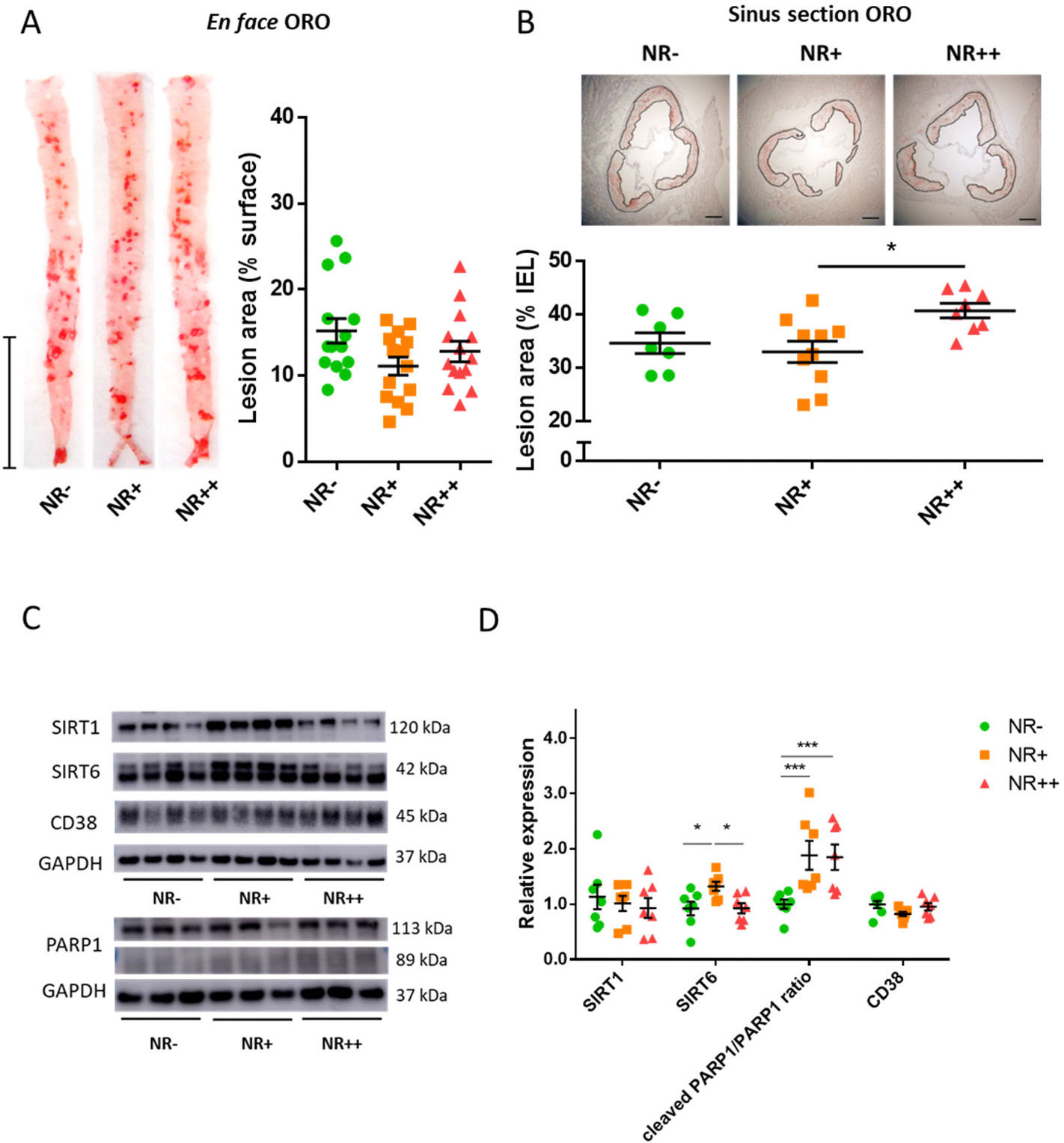
Aortic atherosclerosis in *Apoe* knockout mice fed a high-cholesterol diet supplemented with increasing NR dosage. **(A)** *en face* surface analyses of plaques in thoracoabdominal aortae, stained by Oil Red O (N = 14/group, bar = 1 cm). **(B)** Serial aortic sinus cross sections stained with Oil Red O. Plaque lesion area was selected as indicated and was corrected for total cross-sectional area of Internal Elastic Lamina (IEL) area (N = 7–11/group, bar = 200 μm). **(C)** Representative Western blot images of SIRT1, SIRT6, PARP1, CD38 and GAPDH of aortic lysates; **(D)** Quantitative results of blot images. Expression levels are corrected with GAPDH expression levels. Cleaved PARP1/PARP1 ratio is calculated by the different intensity of PARP1 band at different molecular weights; N = 7/group. Lines indicate mean and SEM for each group. Significance is determined by one-way ANOVA and Tukey’s *post hoc* correction. *, *p* < 0.05; **, *p* < 0.01; ***, *p* < 0.001. ORO, Oil Red O; SIRT, sirtuin; PARP, poly (ADP-ribose) polymerase; CD, cluster of differentiation; GAPDH, glyceraldehyde-3-phosphate dehydrogenase. (For interpretation of the references to colour in this figure legend, the reader is referred to the Web version of this article.)

*En face* analyses of thoraco-abdominal aortae were carried out after excising and opening the aorta longitudinally; aortic roots were analyzed in 4–6 serial equidistant (50 μm) cross sections as described [17]. For the latter, hearts and aortae were embedded in optimal cutting temperature (OCT) and kept frozen at −80 °C. Serial cross sections of 5 μm thickness of the aortic roots were collected. Atherosclerotic plaques were visualized by Oil Red O (ORO) staining. Pictures of stained aortae and sinus sections were quantified by Image J. Plaque lesion area were identified, quantified, and corrected to the whole aorta surface area (*en face*) or internal elastic lamina area (IEL area in sinus sections), (Fig. 1A and B).

### 2.4. Plasma cytokine and lipid biochemical analyses

Plasma was aliquoted and stored at −80 °C until analysis. Cytokines were analyzed using ProcartaPlex Simplex (ICAM-1) or multi-Plex (IFN-γ, TNFα, and IL-6) ELISA method (ThermoFisher) according to the instruction of the manufacturer, read by Luminex MAGPIX Instrument (ThermoFisher). For lipids, before HDL-c (high density lipoprotein-cholesterol) measurement, Apo-B containing lipoproteins were removed from plasma by PEG (polyethylene glycol) precipitation. Total cholesterol and HDL content were measured using cholesterol assay 294–65801 kit (Fujifilm Wako Lab). Triglycerides were measured by TRIGB 11877771 kit (Roche). Free fatty acids (FFA) were measured using NEFA (non-esterified fatty acid) assay 91696 and 91096 kits (Fujifilm Wako Lab). The absorbance of the samples was read through Epoch reader at indicated wavelengths (Cholesterol: 600/700 nm; TG: 505/700 nm; NEFA: 546/600 nm). LDL-c (low-density lipoprotein-cholesterol) was derived from Friedewald Equation.

### 2.5. NAD^+^ quantification and protein quantification

NAD^+^ levels were determined by EnzyChrom^™^ NAD^+^ Assay Kit (E2ND-100, BioAssay), and experimental procedures were carried out as suggested by the manufacturer. Briefly, 50 mg of liver sample or 40 μL plasma was used. Liver samples were homogenized in Extraction Buffer and part of the solution was used to determine the protein concentration by Protein Assay Dye (#5000006, BIO-RAD). Extracted samples were then neutralized by Opposite Extraction Buffer and added to Reagent Solution. A565 was measured after 15 min.

### 2.6. 4PY and 2PY quantification in plasma

Samples were measured in duplicates and blinded to the experimenter. Stable-isotope-dilution LC–MS/MS was used for quantitation of 4PY (*N*^1^-methyl-4-pyridone-3-carboxamide) and 2PY (*N*^1^-methyl-2-pyridone-5-carboxamide) in mouse plasma as previously described [14].

### 2.7. Protein expression levels

Western Blot analyses of tissue lysates and RAW264.7 cell lysates were performed according to standard protocols. Aortae were homogenized with microbeads (P000912-LYSK0-A, Precellys) and liver pieces were crushed with a piston. Electrophoresis-separated proteins were transferred on PVDF membranes (Millipore). Membranes were then incubated with different primary antibodies and host species-specific secondary antibodies (Southern Biotech, 1:10,000). Signals were developed with Immobilon HRP substrate (Millipore). Protein levels were normalized to levels of GAPDH and quantified by Image J (National Institutes of Health, USA). The following antibodies were used: mouse-anti-GAPDH (Merck Millipore, 1:10,000), rabbit-anti-SIRT1 (9475, Cell Signaling Technology, 1:500), rabbit-anti-SIRT6 (12486, Cell Signaling Technology, 1:1000), mouse-anti-PAR (ALX-804–220-R100, ENZO Lifesciences, 1:500), rabbit-anti-PARP-1 (ALX-210–619-R100, ENZO Lifesciences, 1:2000), rabbit-anti-CD38 (92457, Cell Signaling Technology, 1:1000), rabbit-anti-LDLR (MA5–32075, Invitrogen, 1:1,1000), rabbit-anti-LRP1 (26387, Cell Signaling Technology, 1:1,1000), and rabbit-anti-PCSK9 (ab31762, Abcam, 1:1,1000). Signals were detected using Amersham Imager 600 (GE).

### 2.8. Flow cytometry

Flow cytometry was used to determine the markers on RAW264.7 and bone marrow cell plasma membrane after treatment. Cells were digested with 0.25% trypsin, collected, and strained through 40 μm cell strainers. The staining mixture was prepared by mixing Biolegend anti-CD86 antibody (APC, 105012; BV650, 105035), anti-CD38 (Alexa Fluor 488, 102714), anti-CD45 (PerCP/Cy5.5, 103131), anti-CD11b (Pacific Blue, 101224), anti-Ly6C (BV570, 128029), anti-Ly6G (PE/Cy7, 127617) and Fc Receptor blocker (14–9161-73, Thermo Fisher) in a ratio of 1:100 with FACS buffer. The staining mixture (100 μL) was added and incubated at 4 °C for 1 h, shielded from light. Fluorescence intensity was measured using a Cytek^®^ Aurora flow cytometer. The threshold of positivity was determined using unstained and single-stained samples. Data were analyzed with Cytek built-in software or with Floreada.io.

### 2.9. Liver section staining and imaging

Livers were embedded in OCT and frozen at −80 °C. Thawed serial sections were washed with PBS twice and then fixed with 4% paraformaldehyde for 15 min. Sections were blocked with blocking solution (5% goat serum and 5% bovine serum albumin in PBST (PBS+0.1% Tween-20)) overnight at 4 °C in a humid chamber. On the second day, primary antibodies were applied (rabbit-anti-CD38, 1:100, 68336, Cell Signaling; rat-anti-F4/80, 1:100, 14–4801-85, Thermo Fisher; rat-anti-CD86, 1:400, 942-MSM-1, Thermo Fisher) for 3 h at RT in a humid chamber. Sections were washed and blocked again with blocking solution for 1 h RT. Secondary antibodies were applied to the sections (goat-anti-rabbit, 1:5000, A11010, Invitrogen and goat-anti-rat, 1:5,000, A11006, Invitrogen) for 30 min. DAPI were added in the last 5 min. Sections were washed and mounted before visualizing with fluorescence microscopy (Zeiss Axio Observer Z1). Images were quantified by Image J.

### 2.10. Statistical analyses

Different groups were compared using Kruskal–Wallis tests or one-way ANOVA, followed by Tukey *post hoc* comparison where appropriate. The significance level was set at *p* = 0.05. *, *p* < 0.05; **, *p* < 0.01; ***, *p* < 0.001; ****, *p* < 0.0001. Analyses were completed with inbuilt packages of Prism GraphPad. Graphical abstract was created with BioRender.

## 3. Results

### 3.1. High-dose NR supplementation was not atheroprotective

We supplemented different doses of NR (NR−, no addition; NR+, 1.2 g/kg diet; NR++, 2.4 g/kg diet) as NAD^+^ booster in a high-cholesterol diet and analyzed progression of atherosclerosis in *Apoe*^−*/*−^ mice after 12 weeks of treatment. The NR doses were selected based on previous literature of metabolic studies in mice [10]. *En face* analyses of thoracoabdominal aortae showed no significant difference in plaque lesion area (Fig. 1A). Yet, we observed that NR++ mice had increased plaque lesion area in serial cross sections of aortic roots (Fig. 1B).

### 3.2. NR supplementation did not affect body weight

NR++ had higher relative liver weight than NR+ (Supplementary Figs. 1A–C). Blood cell counts were not affected by NR supplementation (Supplementary Figs. 1D–H). Expression of NAD^+^-consuming enzymes in aortic lysates revealed that SIRT6 levels were increased in the NR+ group but decreased in NR++ mice; cleaved PARP-1/total PARP1 ratio was higher in NR+ and NR++. (Fig. 1C–D).

### 3.3. High-dose NR raised pro-inflammatory cytokines, LDL-cholesterol and triglycerides in plasma

**Fig. 2.**
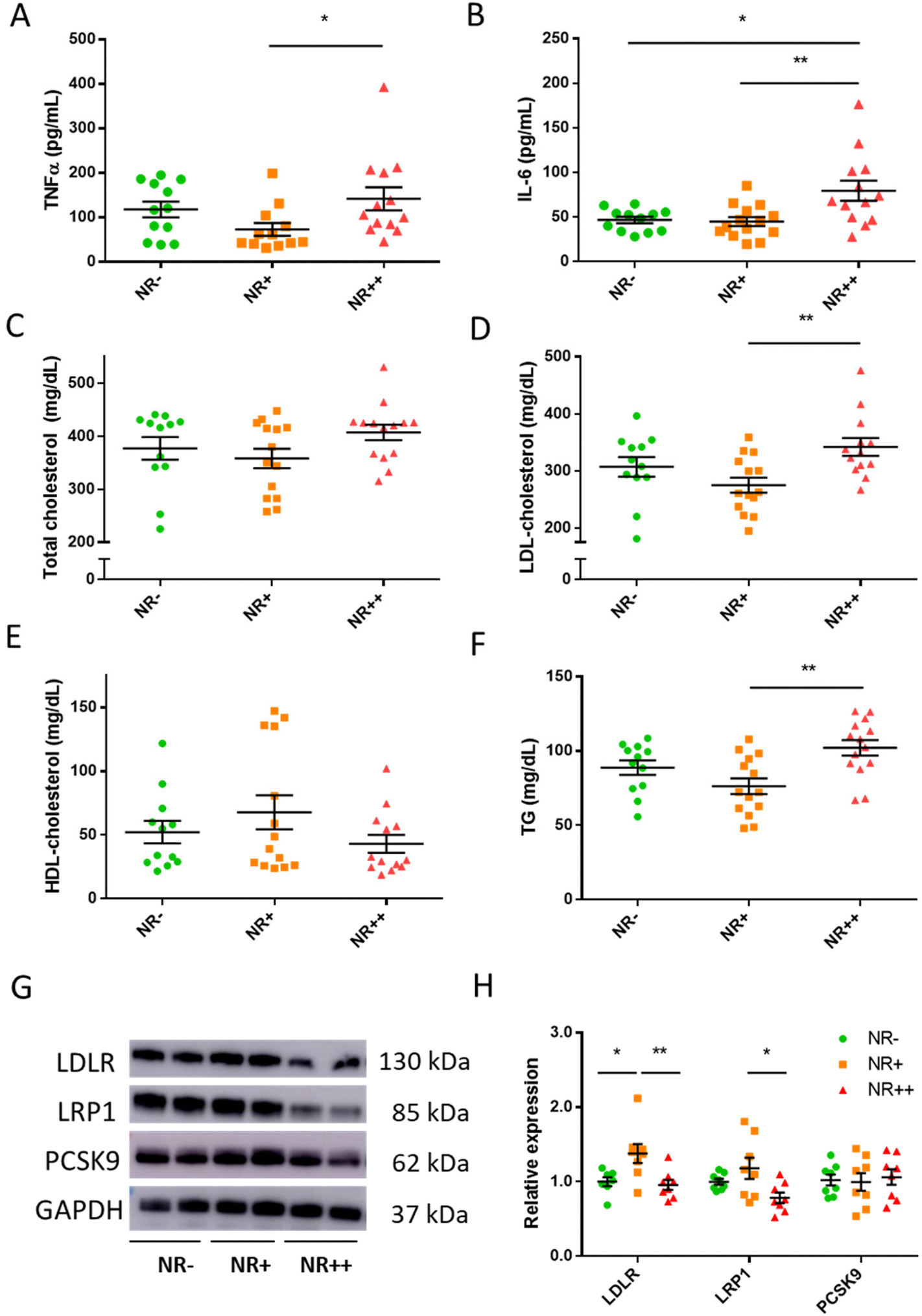
Analyses of plasma cytokines, and lipids in *Apoe* knockout mice fed a high-cholesterol diet supplemented with increasing NR dosage. **(A)** TNFα; (**B)** IL-6; (**C)** Total cholesterol; (**D)** LDL-cholesterol; (**E**) HDL-cholesterol; **(F)** triglyceride (TG) in plasma of *Apoe* knockout mice supplemented with different doses of NR. N = 12–13/group. **(G)** Western blot images of LDLR, LRP1, PCSK9 and GAPDH of liver lysates; **(H)** Quantitative results of blot images. Expression levels are corrected for GAPDH expression levels. N = 7–8/group. Individual datapoints are shown in the figure, and the lines indicate mean and SEM for each group. Significance is determined by one-way ANOVA and Tukey’s *post hoc* correction. *, *p* < 0.05; **, *p* < 0.01. LDLR, low-density lipoprotein receptor; LRP1, Low-density lipoprotein receptor-related protein 1; PCSK9, Proprotein convertase subtilisin/kexin type-9.

We have showed that atherosclerosis induced systemic inflammation [18], and we ask if this has been affected by NR supplementation in our mice. Increased systemic inflammation was detected in NR++ with elevated plasma levels of TNFα and IL-6 (Fig. 2A–B). Plasma ICAM-1 and IFN-γ remained unchanged (Supplementary Figs. 2A–B). Effects of NR on lipid metabolism revealed that total cholesterol levels did not differ between groups (Fig. 2C). LDL-c (low-density lipoprotein cholesterol) in NR++ group was the highest, whereas NR supplementation did not change HDL-c (high-density lipoprotein cholesterol, Fig. 2D–E). NR++ mice showed higher plasma triglycerides than the NR+ group (Fig. 2F), but free fatty acid level did not change upon NR supplementation (Supplementary Fig. 2C). Investigations of liver lipid metabolism revealed that lipoprotein receptors such as LDLR (low-density lipoprotein receptor) and LRP1 (Low-density lipoprotein receptor-related protein 1) were decreased in NR++ group (Fig. 2G–H). Proprotein convertase subtilisin/kexin type-9 (PCSK9), the degrading binding partner of LDLR, did not change upon NR supplementation. Liver cholesterol and triglyceride levels did not change in response to high-dose NR supplementation (Supplementary Figs. 3A–B).

### 3.4. NR supplementation increased terminal NAD^+^ metabolites in plasma

**Fig. 3.**
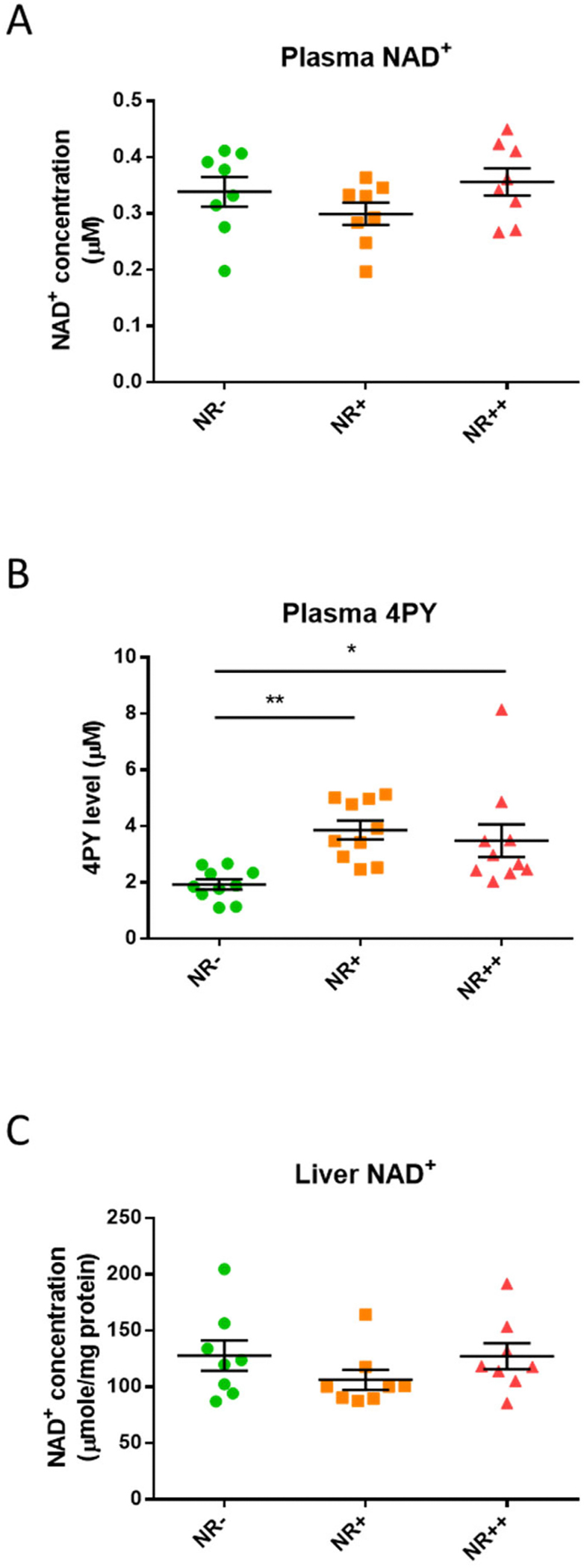
NAD^+^ concentrations and downstream metabolite 4PY in *Apoe* knockout mice fed a high-cholesterol diet supplemented with increasing NR dosage. NAD^+^ concentration in **(A)** plasma and in **(C)** liver lysates. N = 8/group. (**B)** 4PY levels in plasma. N = 10/group. Individual datapoints are shown in the figure, and the lines indicate mean and SEM for each group. Significance is determined by one-way ANOVA and Tukey’s *post hoc* correction. *, *p* < 0.05; **, *p* < 0.01. 4PY, *N*^1^-methyl-4-pyridone-3-carboxamide.

We examined the efficacy of NR supplementation under atherosclerotic environment. Plasma NAD^+^ concentration did not increase in this setting, while the NAD^+^ downstream metabolites 4PY and 2PY increased upon NR supplementation (Fig. 3A–B and Supplementary Fig. 4A). The two metabolites were highly correlated with each other (Supplementary Fig. 4B). NAD^+^ concentrations in liver lysates did not change (Fig. 3C). On the contrary, the same dose has been reported effective in other studies in boosting NAD^+^ [11]. Taken together, we suspected that under pro-inflammatory, atherosclerotic conditions, supplemented NAD^+^ was consumed.

### 3.5. High-dose NR increased the NAD^+^-consuming CD38 in liver lysates of atherosclerotic mice

**Fig. 4.**
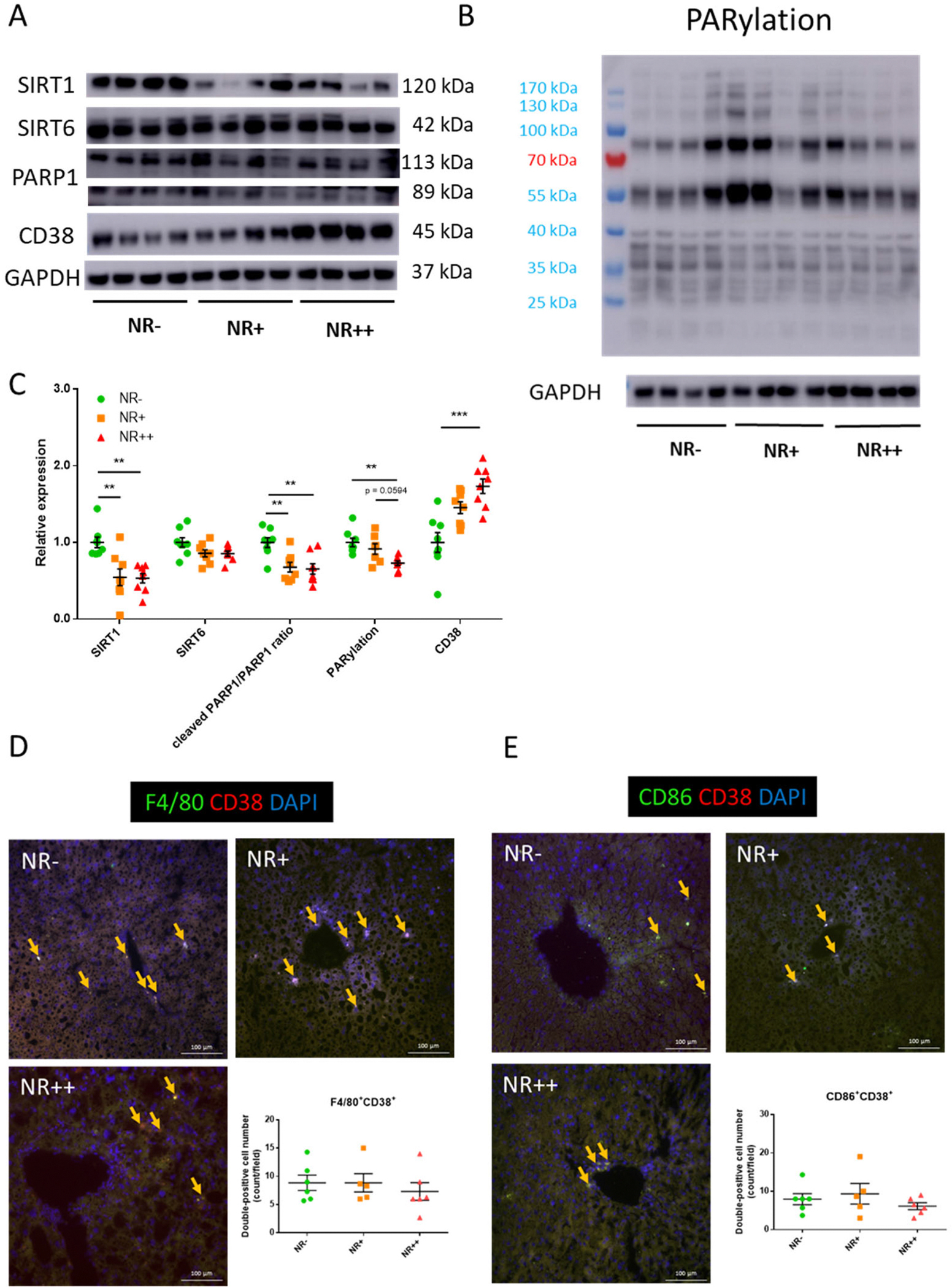
Expression levels of NAD+-consuming enzymes in livers of *Apoe* knockout mice fed a high-cholesterol diet supplemented with increasing NR dosage. **(A)** Western blot images of SIRT1, SIRT6, PARP1, CD38 and GAPDH of liver lysate; **(B)** Western blot image of overall PARylation of proteins and GAPDH in liver lysate; (**C)** Quantitative results of blot images. Expression levels are corrected with GAPDH expression levels. Cleaved PARP1/PARP1 ratio is calculated by the different intensity of PARP1 band at different molecular weights; N = 8/group. (**D)**&(**E),** Representative photomicrographs with quantification of immunofluorescence staining of F4/80, CD86, and CD38 on liver sections (bar = 100 mm). Yellow arrows indicate double positivity. Individual datapoints are shown in figure, lines indicate mean and SEM for each group. Significance is determined by one-way ANOVA and Tukey’s *post hoc* correction. *, *p* < 0.05; **, *p* < 0.01; ***, *p* < 0.001. SIRT, sirtuin; PARP, poly (ADP-ribose) polymerase; CD, cluster of differentiation; GAPDH, glyceraldehyde-3-phosphate dehydrogenase; PARylation, poly(ADP)-ribosylation. (For interpretation of the references to colour in this figure legend, the reader is referred to the Web version of this article.)

As we observed changes in lipids, we checked liver for NAD^+^-metabolizing enzymes. We investigated protein expression levels of NAD^+^-consuming enzymes in liver lysates: SIRT1, SIRT6, CD38, PARP1 and PARylation pattern as PARP activity. SIRT1, cleaved PARP1/total PARP ratio, and PARylation decreased in NR-supplemented groups, suggesting lower PARP activities (Supplementary Fig. 5A). On the other hand, CD38 levels increased in NR++ liver lysates (Fig. 4A–C).

These data suggest that liver CD38 consumes NAD^+^ in an atherosclerotic environment when supplemented with NR. The numbers of liver macrophages did not change upon NR supplementation (Fig. 4D–E).

### 3.6. High-dose NR increased CD38 expression in oxLDL-treated RAW264.7 macrophages

To further unveil the contribution of macrophages, we employed an *in vitro* model mimicking the context of atherosclerosis. Thereby, we stimulated mouse macrophage cell line RAW264.7 with oxLDL (human oxidized LDL) and examined protein expression levels of NAD^+^-consuming enzymes.

**Fig. 5.**
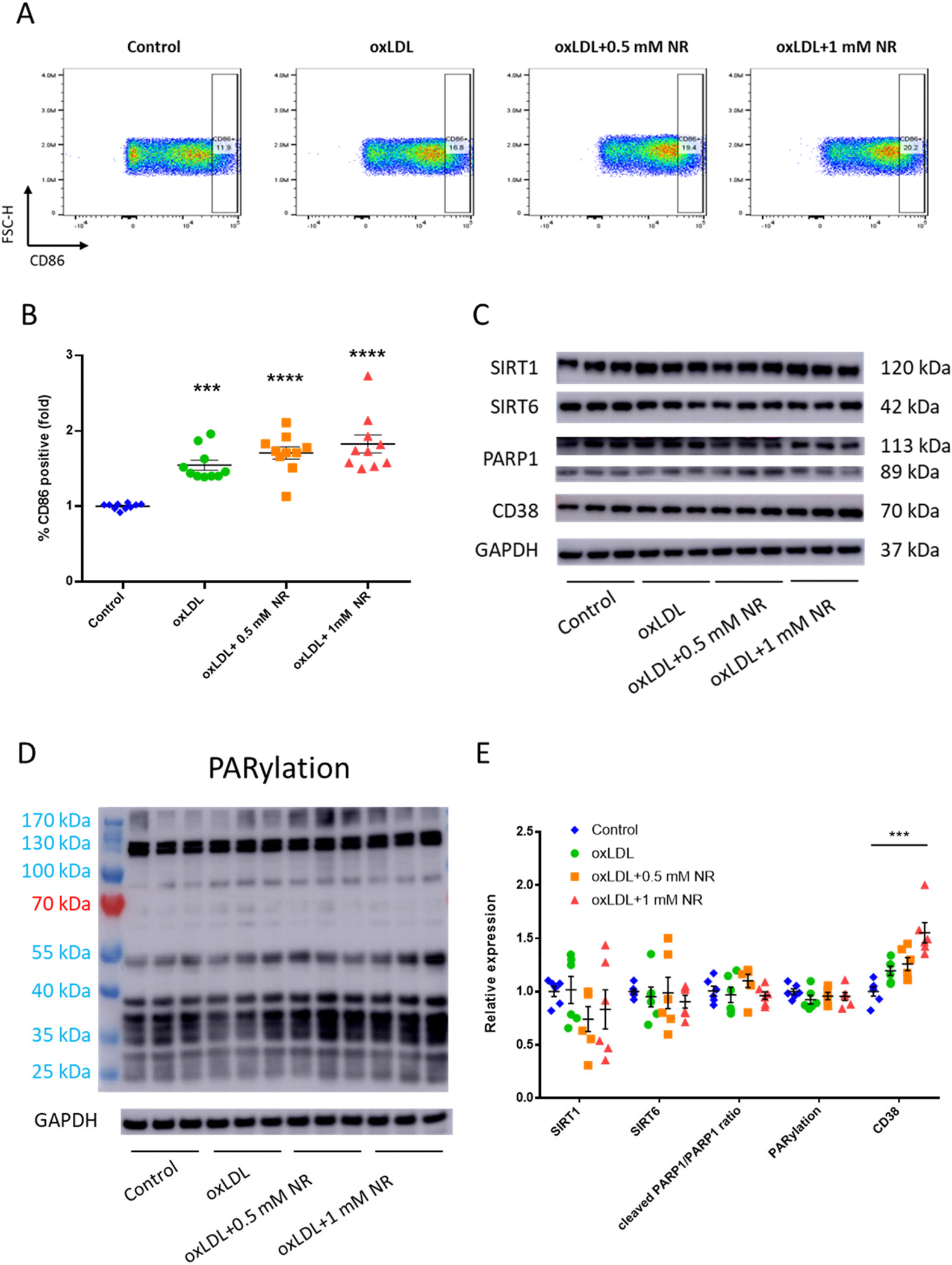
Protein expression levels of NAD^+^-consuming enzymes in oxLDL-treated RAW264.7 macrophages upon increasing exogenous NR. **(A)&(B)** Flow cytometric analyses of expression of CD86 of *RAW* cells stimulated with atherosclerotic stimuli and NR. N = 9/group. **(C),** representative Western blot images of SIRT1, SIRT6, PARP1, CD38 and GAPDH of *RAW* cell lysate; **(D)** Representative Western blot image of overall PARylation of proteins and GAPDH in *RAW* cell lysate; **(E)** Quantitative results of blot images. Expression levels are corrected for GAPDH expression levels. Cleaved PARP1/PARP1 ratio is calculated by the different intensity of PARP1 band at different molecular weights; N = 6/group. Individual datapoints are shown in the figure, and the lines indicate mean and SEM for each group. Significance is determined by one-way ANOVA and Tukey’s *post hoc* correction. *, *p* < 0.05; **, *p* < 0.01; ***, *p* < 0.001, ****, *p* < 0.0001. SIRT, sirtuin; PARP, poly (ADP-ribose) polymerase; CD, cluster of differentiation; GAPDH, glyceraldehyde-3-phosphate dehydrogenase; PARylation, poly(ADP)-ribosylation.

After treatment of oxLDL with/without NR, cell viability was unperturbed (Supplementary Figs. 6A–B). *RAW* cells also tended to be more pro-inflammatory and expressed CD86 when treated with atherosclerotic stimuli, which NR supplementation did not suppress (Fig. 5A–B). CD38 is the only NAD^+^-consuming enzyme which increased in high NR group, albeit with a shift in molecular weight (Fig. 5C–E), possibly due to dimerization reported in immune cells in previous literature [19,20] (Supplementary Figs. 6C–D). Others have reported that macrophages express more CD38 when activated [21]. There was no change in PARP1 cleaved ratio nor in PARylation pattern (Fig. 5C–E). Both full length and cleaved PARP1 expression were lowered upon NR supplementation (Supplementary Fig. 5B).

### 3.7. High-dose NR increased CD38 and CD86 expression in oxLDL-treated bone marrow macrophages

**Fig. 6.**
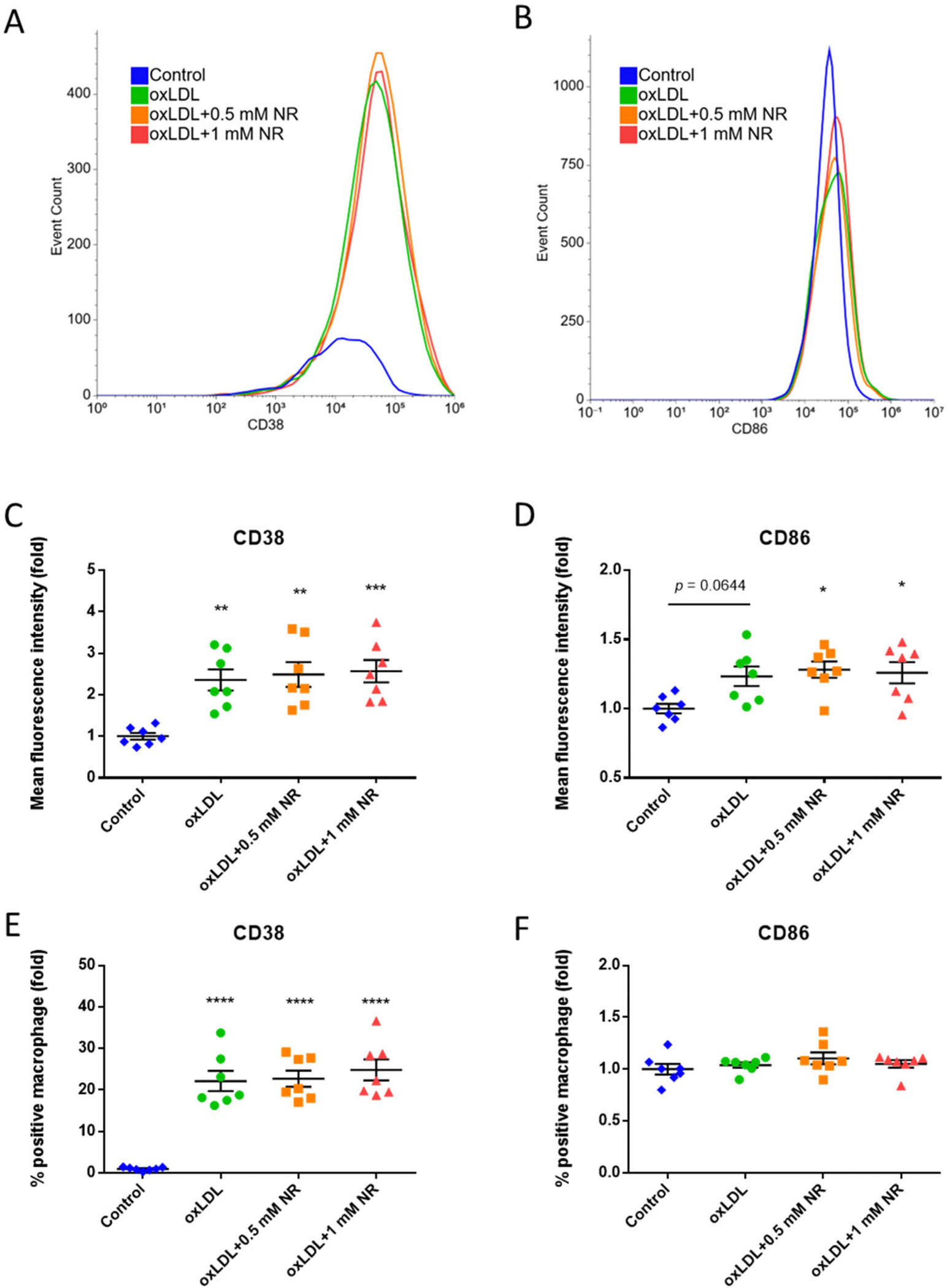
CD38 and CD86 analyses in bone marrow macrophages treated with oxLDL upon increasing exogenous NR. Flow cytometric analyses of expression of **(A)** CD38 & **(B)** CD86 of bone marrow-derived macrophages stimulated with oxLDL and NR. Cells were gated by CD45^+^ CD11b^+^ Ly6C^+^ Ly6G^−^ for macrophages. Representative expression pattern of CD38, CD86 are shown. N = 7/group. Geometrical mean of fluorescence intensity of **(C)** CD38 & **(D)** CD86 of gated macrophages and percentage of positive cells for **(E)** CD38 & **(F)** CD86. Numbers were indexed by the means of the control group. N = 7/group. Individual data points are shown in the figure, and the lines indicate mean and SEM for each group. Significance is determined by one-way ANOVA and Tukey’s *post hoc* correction. *, *p* < 0.05; **, *p* < 0.01; ***, *p* < 0.001, ****, *p* < 0.0001 compared to control group. CD, cluster of differentiation.

We then isolated bone marrow cells and stimulated them with the same setup to investigate whether primary macrophages behaved similarly. We gated the macrophages from bone marrow cells and examined the expression levels of CD38 and CD86. We showed that oxLDL stimulation increased the level of CD38, while both NR dosages did not rescue it (Fig. 6A and C). As for CD86, oxLDL alone was not sufficient to increase CD86 levels. However, along with NR, there was a significant increase of CD86 compared to the control group (Fig. 6B and D). oxLDL and NR markedly increased the percentage of CD38-positive macrophages, but did not change the ratio of CD86-positive cells (Fig. 6E–F).

## 4. Discussion

**Fig. 7.**
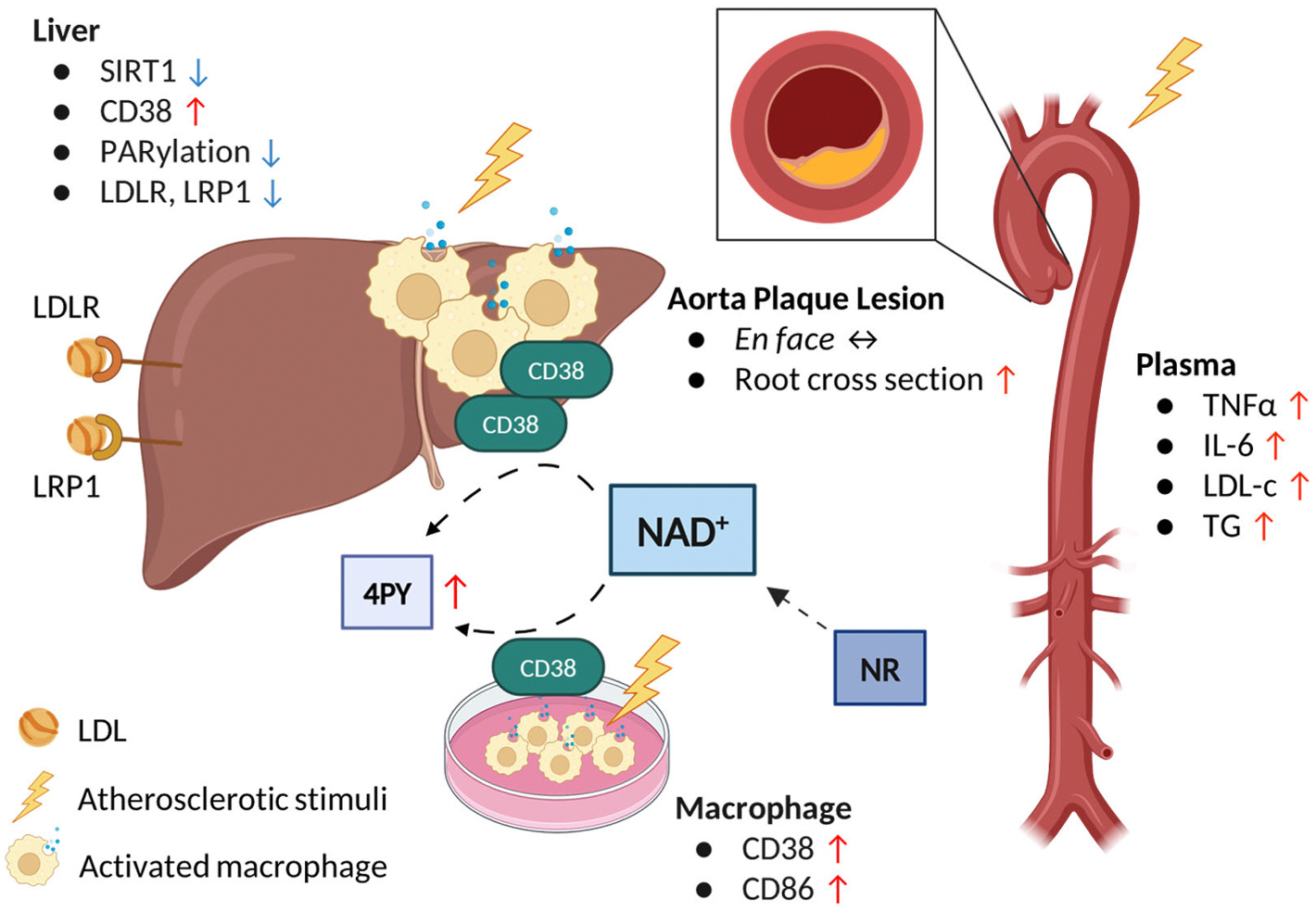
Supplementation of NR as NAD^+^-boosting strategy did not show protective effects in atherosclerosis-prone *Apoe*-deficient mice. After 12 weeks of NR supplementation, we observed that high-dose NR treatment modestly increased area in aortic root cross sections and increased systemic inflammation and plasma lipids and the downstream metabolite 4PY. With high NR supplementation, decreased levels of lipoprotein receptors were observed in liver lysates, along with decreased SIRT1 expression and PARP activity. CD38 was increased as one of the NAD^+^ -consuming enzymes in liver lysate, RAW264.7 macrophage cell line, and bone marrow macrophages. CD86 expression increased in bone marrow macrophages stimulated with oxLDL and high NR. *Apoe*, apolipoprotein E, CD, cluster of differentiation; IL, interleukin; LDL-c, low-density lipoprotein cholesterol; LDLR, low-density lipoprotein receptor; LRP1, Low-density lipoprotein receptor-related protein 1; NAD^+^, nicotinamide adenine dinucleotide; NR, nicotinamide riboside; PARP, poly (ADP-ribose) polymerase; PARylation, poly(ADP)-ribosylation; 4PY, *N*^1^-methyl-4-pyridone-3-carboxamide; SIRT, sirtuin; TNF, tumor necrosis factor; TG, triglycerides.

Our results showed that using increasing dosages of dietary NR to boost NAD^+^ levels did not provide atheroprotection in mice. In the high-dose NR group, plaque lesions in aortic root cross sections and systemic pro-inflammatory cytokine levels were even increased, and LDL-cholesterol, triglycerides and the terminal NAD^+^ metabolites 4PY and 2PY also rose. In parallel, with high-dose NR, increased hepatic levels of the NAD^+^-consuming enzyme CD38 were observed. Cultured oxLDL-treated *RAW* macrophages also expressed more CD38 and pro-inflammatory markers upon stimulation with increased dosages of NR (Fig. 7).

Genetic loss-of-function studies have shown that sirtuins, especially SIRT1 and SIRT6 provide important protection in atherosclerotic mice [1]. Heterozygous genetic deletion of SIRT1 increased plaque area in *Apoe*^−/−^ mice upon high-cholesterol diet [17]. SIRT6-deficient bone marrow cells enhanced plaque area in *Apoe*^−/−^ mice, likely caused by increased macrophage scavenger receptor expression. These findings suggest an immune regulating and atheroprotective role of SIRT6 [22].

Pharmacologically, we have demonstrated that a specific SIRT1 activator conferred beneficial effects in preclinical atherosclerosis models [4]. Individually, increased expression or pharmacological activation of SIRT1/SIRT6 was beneficial for combatting atherosclerosis [23,24]. Yet, our current findings revealed that the strategy aimed to activate all sirtuins using NAD^+^ boosting did not show the expected protection. It seems that the effects of NAD^+^ boosting are dependent on the context, particularly in cardiovascular diseases [1,3].

In liver lysates and in cultured macrophages, high-dose NR increased CD38 levels, which remained the only NAD^+^-consuming enzyme that was induced. We suspect that high-dose NAD^+^ boosting shifts NAD^+^ metabolism towards activation of the CD38 pathway which may account in part for the lack of beneficial effects in our atherosclerosis model. Notably, increased CD38 levels have been reported in oxLDL-treated macrophages exposed to niacin [25]. In hypertensive patients, increased IL-1β levels were associated with elevated expression of CD38, thus exhausting available NAD^+^ [26]. Other studies related the decrease of NAD^+^ upon aging to increased expression of CD38 in pro-inflammatory macrophages [21,27] or to PARP consumption [28]. Given these reports and our findings, we inferred that CD38 is an important NAD^+^-consuming enzyme in pathological conditions. Its association with NAD^+^ levels needs to be carefully monitored.

Originally, supplements like NR and other NAD^+^ precursors were thought to be beneficial to activate all sirtuins and garner their positive effects. Meanwhile, an increasing number of studies revealed that the effects of boosting NAD^+^ at high dosages depend on the context. Notably, they may not be protective and even cause harm in certain conditions. At the experimental level, NR supplementation reduced atherosclerotic plaques and improved endothelial function in *Apoe* knockout mice [29], but it did not show protective effects in LDLR (LDL-receptor) knockout mice [30]. NR added to a high-fat diet for 18 weeks enhanced macrophage infiltration in the adipose tissue and increased inflammation markers. This in turn induced hyperinsulinemia and deteriorated glucose tolerance of these mice [31]. Moreover, in an acute inflammatory context of LPS (lipopolysaccharide) stimulation, NR treatment further increased pro-inflammatory cytokines; suppressing NAD^+^ synthesis reversed this phenomenon [32]. A slightly more potent analog of NR, NRH (dihydronicotinamide riboside) [33], also enhanced inflammation in mouse and human macrophages [34]. Therefore, NAD^+^ boosting may in fact result in pro-inflammatory effects. Our results complement these findings in the context of atherosclerosis, showing that supplementing high-dose NR increased inflammation.

A recent series of clinical and mechanistic studies revealed that increased circulating levels of the NAD^+^ downstream metabolites 2PY and 4PY, which are only produced when the NAD^+^ pool is replete, are associated with heightened risk for incident (3 year) major adverse cardiovascular events. Moreover, the structural isomer 4PY, but not 2PY, directly fostered vascular inflammation including enhanced expression of VCAM-1 and leukocyte-endothelial cell interactions [14]. Our study confirmed that supplementing NAD^+^ precursors increased 4PY and 2PY concentrations *in vivo*. This may explain in part the increased plaque lesions in aortic root cross section in the high-dose NR group. In addition, we have applied longer NR treatment periods compared to other studies that investigated endothelial function and atherosclerosis in mice [29,35]. It is conceivable that high-dose NR supplementation over an extended treatment period led to an overflow of the NAD^+^ pool and accumulation of pro-inflammatory end-products. Of note, concentrations of key metabolites may be different across species. For example, plasma 4PY concentrations were higher in mice than in humans.

Moreover, we demonstrated that SIRT6 and PARP1 expression patterns depend on the tissue context. In aortic lysates, SIRT6 and cleaved PARP1 increased upon low-dose NR supplementation and SIRT6 decreased upon high-dose NR. We have shown that knock-down of SIRT6 in human aortic endothelial cells increased cleaved PARP1 [36]. It is known that increased PARP1 is associated with pro-inflammatory factors in atherosclerosis [37]. Thus, these findings match the increased aortic plaque lesions in this current study.

### 4.1. Potential limitations

It is important to note that the differences in energy metabolism between mice and humans limit extrapolations from mice to humans. Additionally, determining the optimal dose of NR supplementation in our model is challenging, and restricted to a proof-of-principle. We did not measure food intake in this study, but body weights did not differ between the study groups at any time point and previous studies using the same dose of NR supplementation did not affect food intake [10,11]. Moreover, it is not possible to determine sirtuin or CD38 activity after supplementation with NR. Sirtuins can deacetylate multiple targets and CD38 can produce different metabolites. We used protein expression levels as a proxy.

## 5. Conclusions

Our study on atherosclerotic mice supplementing NR as a NAD^+^ boosting strategy showed no protective effects in atherosclerosis. High NR supplementation even increased atherosclerotic plaques in aortic roots, plasma LDL-c, TG, and pro-inflammatory cytokines, which may be mediated by increased CD38 levels and 4PY (Fig. 7).

The AIM-HIGH trial [12] and a recent meta-analysis support this notion (i.e. paradoxical heightened risk with niacin supplementation) [14]. Thus, although NAD^+^ boosting has gained considerable popularity for promoting longevity and extending health span, caution should be used when supplementing high dosage NAD^+^ precursors in patients with atherosclerosis, or at increased risk for cardiovascular disease. Further pharmacological and metabolomic research is needed to improve our understanding of the effects of NAD^+^ boosting.

## Supplementary Material

Wang et al suppl 1

Wang et al suppl 3

Wang et al suppl 2

Wang et al suppl 4

Appendix A. Supplementary data

Supplementary data to this article can be found online at https://doi.org/10.1016/j.atherosclerosis.2025.119188.

## Abbreviations

4PY: *N*^1^-methyl-4-pyridone-3-carboxamide
Apoe: Apolipoprotein E
CD: Cluster of differentiation
EDTA: Ethylenediaminetetraacetic acid
FACS: Fluorescence-activated cell sorting
FFA: Free fatty acid
GAPDH: Glyceraldehyde-3-phosphate dehydrogenase
HDL: High-density lipoprotein
IEL: Internal elastic lamina
IL: Interleukin
LC–MS/MS: Liquid chromatography-tandem mass spectrometry
LDL: Low-density lipoprotein
LDLR: Low-density lipoprotein receptor
LRP1: Low-density lipoprotein receptor-related protein 1
NAD^+^: Nicotinamide adenine dinucleotide
NEFA: Non-esterified fatty acid
NR: Nicotinamide riboside
oxLDL: Oxidized low-density lipoprotein
PARP1: Poly-[ADP-ribose] polymerase-1
PARylation: Poly(ADP)-ribosylation
PCSK9: Proprotein convertase subtilisin/kexin type-9
SIRT: Sirtuin
TG: Triglycerides
TNFα: Tumor necrosis factor alpha
