# Supplementary material for: NAD+ boosting increases atherosclerotic plaques and inflammation in Apoe knockout mice
Source: Atherosclerosis. Author manuscript; Available in PMC 2026 Apr 3. (PMC12512467; doi:10.1016/j.atherosclerosis.2025.119188)
Supplement: Wang et al suppl 1 [file NIHMS2112546-supplement-Wang_et_al_suppl_1.pdf]

Supplementary Methods

Blood cell counts

EDTA whole blood was aliquoted and then analyzed with Scil Vet abc Plus+ (Scilvet). Parameters including WBC (white blood cell), GRA (granulocyte), LYM (lymphocyte), MON (monocyte), and EOS (eosinophil) are recorded.

Liver lipid measurements

Liver lipids were extracted and quantified using commercial kits (Total cholesterol: Invitrogen, EEA026 and Triglycerides: Abcam, ab65336). The protocols were carried out as kit manufacturers suggested, and lipids were indexed by the liver sample weight. For triglycerides, colorimetric assay was used.

Cell toxicity assay

After treatment, culture medium was removed and cells were incubated with MTT (3-(4,5-dimethylthiazol-2-yl)-2,5-diphenyltetrazolium bromide, M6494, Sigma) solution for 4 hours. MTT working solution (0.5 mg/mL) was prepared freshly and diluted with 10% FBS/DMEM medium. Acidic isopropanol (0.04 N HCl) was added to dissolve the formazan crystals and A570 was measured.
