# Supplementary material for: NAD+ boosting increases atherosclerotic plaques and inflammation in Apoe knockout mice
Source: Atherosclerosis. Author manuscript; Available in PMC 2026 Apr 3. (PMC12512467; doi:10.1016/j.atherosclerosis.2025.119188)
Supplement: Wang et al suppl 3 [file NIHMS2112546-supplement-Wang_et_al_suppl_3.pdf]

Supplementary Figure Legends

Supplementary Figure 1. NR supplementation did not change weight gain, liver weight, and white blood cell counts in *Apoe* knockout mice fed a high-cholesterol diet. (A) growth curve of *Apoe* knockout mice supplemented with different doses of NR. Body weight was measured every 2 weeks. **(B)** (absolute) and **(C)** (relative) liver weight, **(D)** white blood cells, **(E)** granulocyte percentage, **(F),** lymphocyte percentage, **(G)** granulocyte/lymphocyte ratio, **(H)** monocyte percentage, **(I)** eosinophil percentage of white blood cells in *Apoe* knockout mice supplemented with different doses of NR. N= 12-14/group. Lines indicate mean and SEM for each group. Significance is determined by one-way ANOVA and Tukey's post hoc correction. *, $p<0.05$. WBC, white blood cell; GRA, granulocyte; LYM, lymphocyte; MON, monocyte; EOS, eosinophil.

Supplementary Figure 2. NR supplementation did not change IFN-γ, ICAM-1, or FFA in *Apoe* knockout mice fed a high-cholesterol diet. (A) IFN-γ **(B)** ICAM-1 and **(C)** free fatty acid in plasma of *Apoe* knockout mice supplemented with different doses of NR. N= 12-14/group. Lines indicate mean and SEM for each group. Significance is determined by one-way ANOVA. IFN, interferon; ICAM, intercellular adhesion molecule; FFA, free fatty acid.

Supplementary Figure 3. Lipid analyses from liver lysates of *Apoe* knockout mice fed a high-cholesterol diet. (A) Total cholesterol and **(B)** triglycerides. N= 6/group. Lines indicate mean and SEM for each group. Significance is determined by one-way ANOVA and Tukey's post hoc correction. *, $p<0.05$.

Supplementary Figure 4. NR supplementation increased 2PY level in *Apoe* knockout mice fed a high-cholesterol diet. (A) 2PY in plasma of *Apoe* knockout mice supplemented with different doses of NR. N= 10/group. **(B)** 4PY and 2PY expression levels are highly correlated with each other. 2PY, N1-methyl-2-pyridone-5-carboxamide; 4PY, N1-methyl-4-pyridone-3-carboxamide. Lines indicate mean and SEM for each group. Significance is determined by one-way ANOVA and Tukey's post hoc correction and Pearson correlation. *, $p<0.05$; **, $p<0.01$.

Supplementary Figure 5. NR supplementation reduced PARP1 cleavage *in vivo* and *in vitro*. PARP1 full length and cleaved PARP1 expression in **(A)** liver and in **(B)** RAW264.7 macrophage cell lysates**.** N= 6-8/group. Lines indicate mean and SEM. Significance is determined by one-way ANOVA and Tukey's post hoc correction. Individual datapoints are shown in the figure, and the lines indicate mean and SEM for each group. *, $p<0.05$; **, $p<0.01$; ***, $p<0.001$; ****, $p<0.0001$. PARP, poly (ADP-ribose) polymerase; GAPDH, glyceraldehyde-3-phosphate dehydrogenase.

Supplementary Figure 6. RAW cell viability after treatment of oxLDL and NR and CD38 expression levels in liver lysates and in RAW macrophages. (A) RAW264.7 cells were treated with different doses of oxLDL in starvation medium for 24 hrs. Relative cell viability is shown**. (B)** Relative cell viability when macrophages were treated with ox-LDL 25 µg/mL along with different doses of NR. N= 3/group. **(C)&(D)** Representative Western blot images of CD38 and GAPDH. Arrow in **(C)** indicates the shifted band in macrophage cell lysate samples. Lines indicate mean and SEM for each group. Significance was determined by one-way ANOVA and Tukey's post hoc correction. Individual datapoints are shown in the figure. *, $p<0.05$; **, $p<0.01$; ***, $p<0.001$. CD, cluster of differentiation; WT, wild type; KO, knockout; RAW, RAW264.7 macrophage cell line.
