# Supplementary material for: NAD+ boosting increases atherosclerotic plaques and inflammation in Apoe knockout mice
Source: Atherosclerosis. Author manuscript; Available in PMC 2026 Apr 3. (PMC12512467; doi:10.1016/j.atherosclerosis.2025.119188)

Supplementary Figure 1

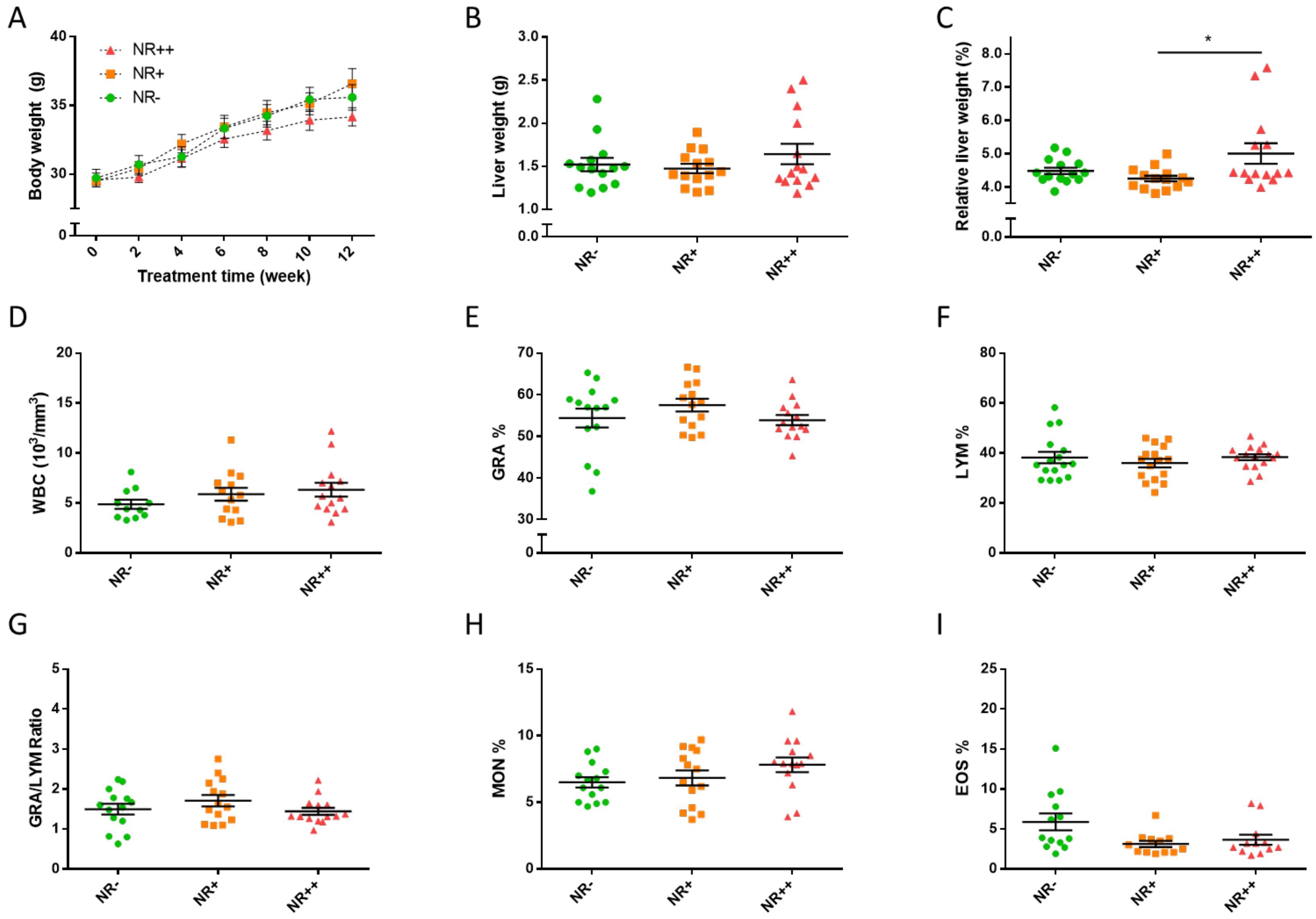

Supplementary Figure 2

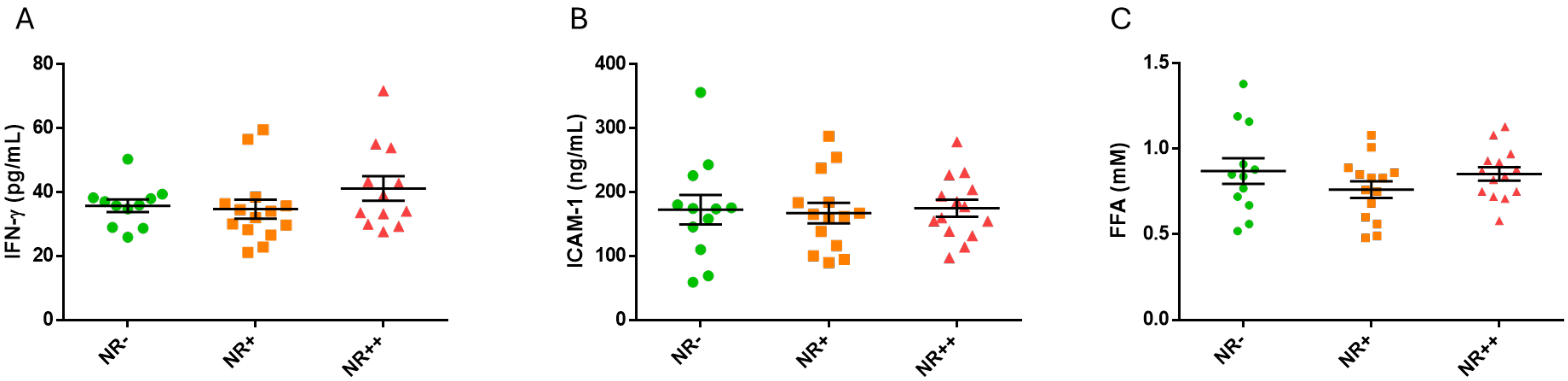

__Supplementary Figure 3__

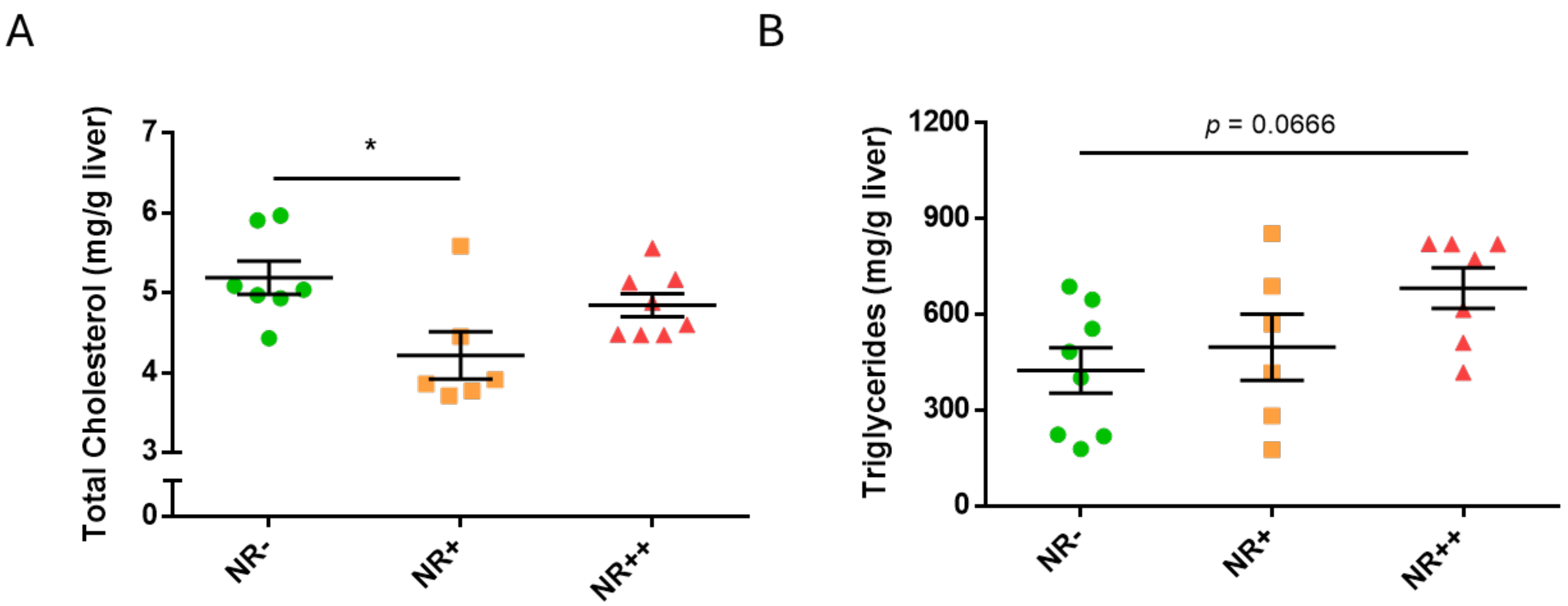

Supplementary Figure 4

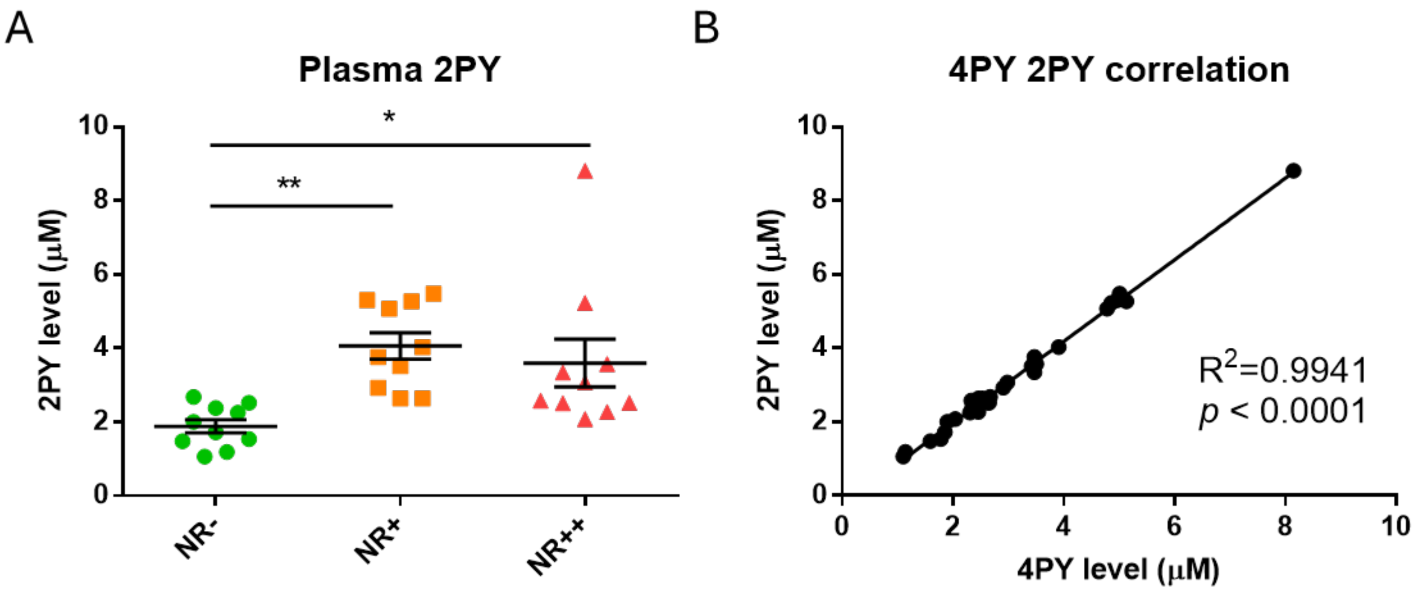

Supplementary Figure 5

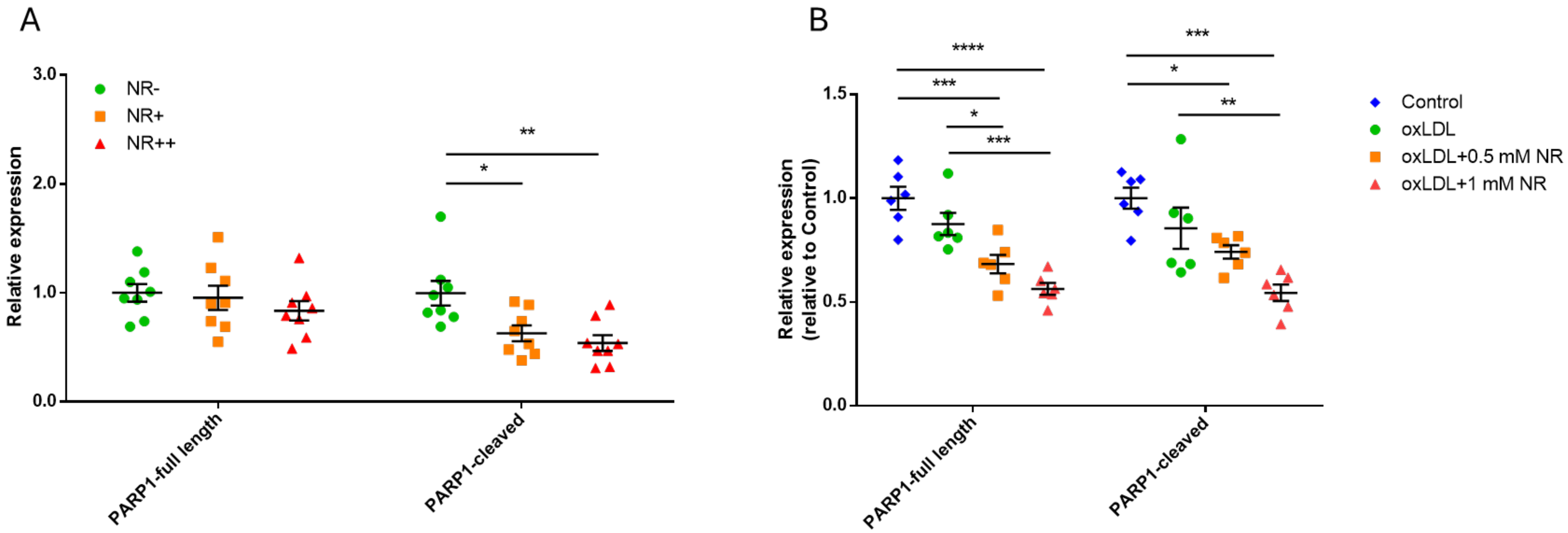

Supplementary Figure 6

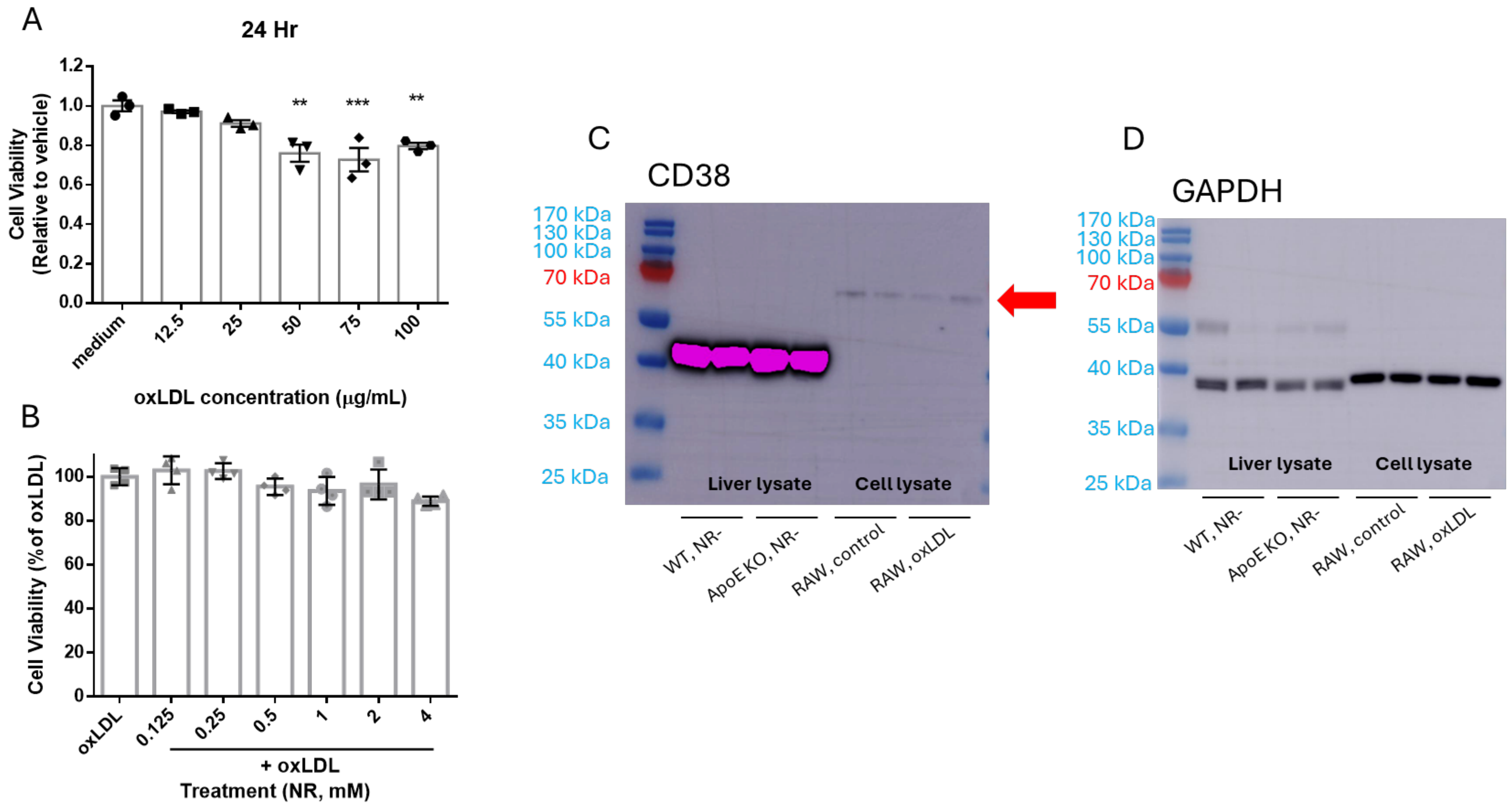

Supplement: Wang et al suppl 2 [file NIHMS2112546-supplement-Wang_et_al_suppl_2.pdf]
