# Supplementary figures and images for: NAD+ boosting increases atherosclerotic plaques and inflammation in Apoe knockout mice
Source: Atherosclerosis. Author manuscript; Available in PMC 2026 Apr 3. (PMC12512467; doi:10.1016/j.atherosclerosis.2025.119188)

Fig. 1C

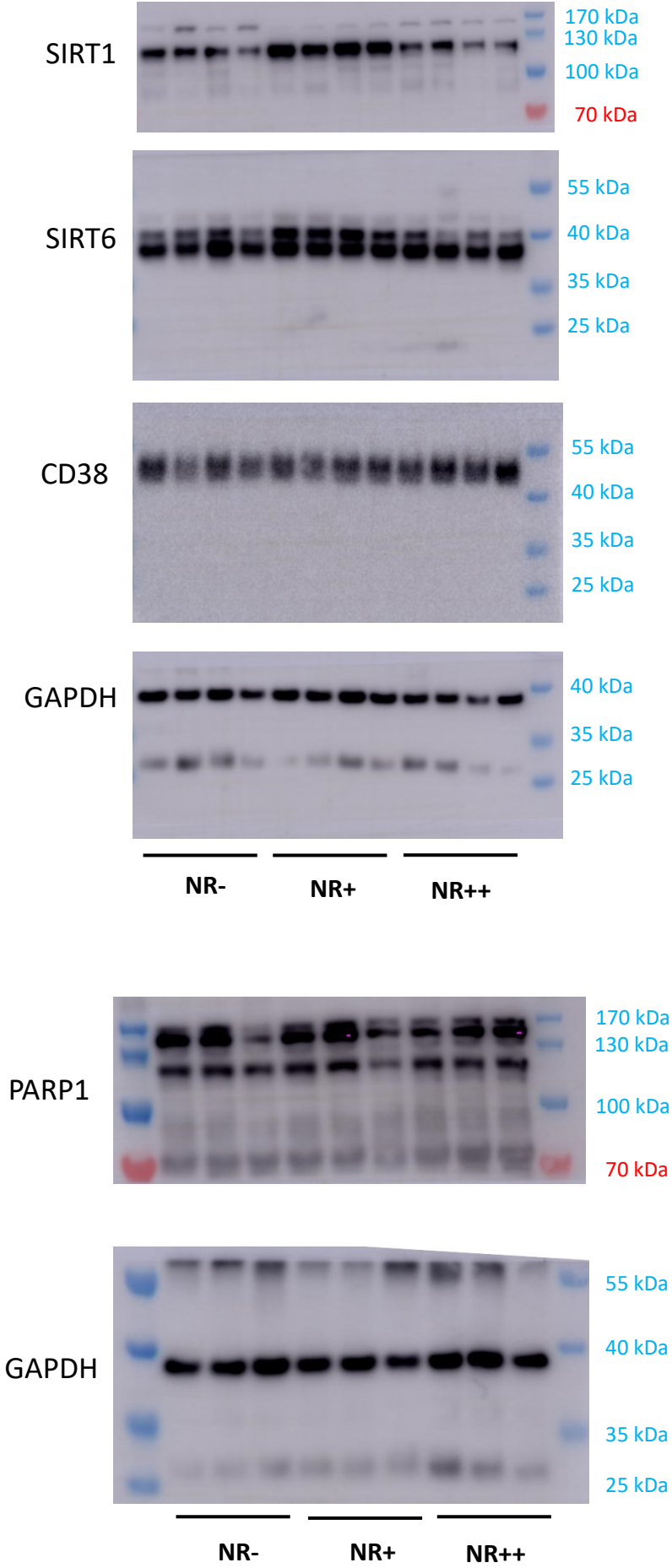

Fig. 2G

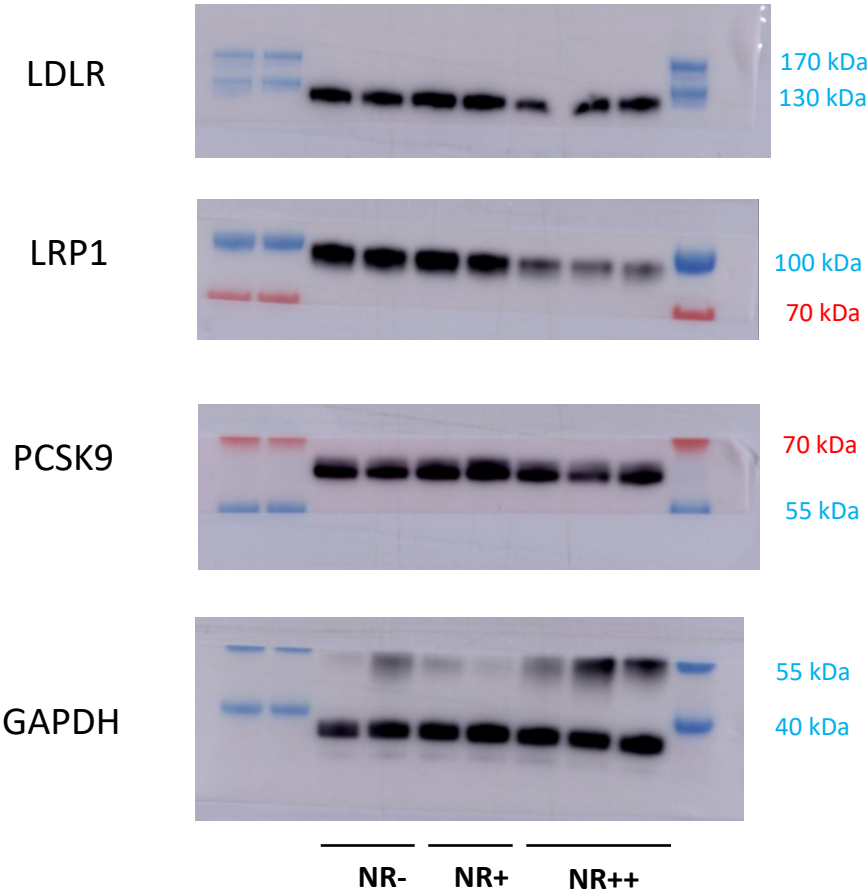

Fig. 4A

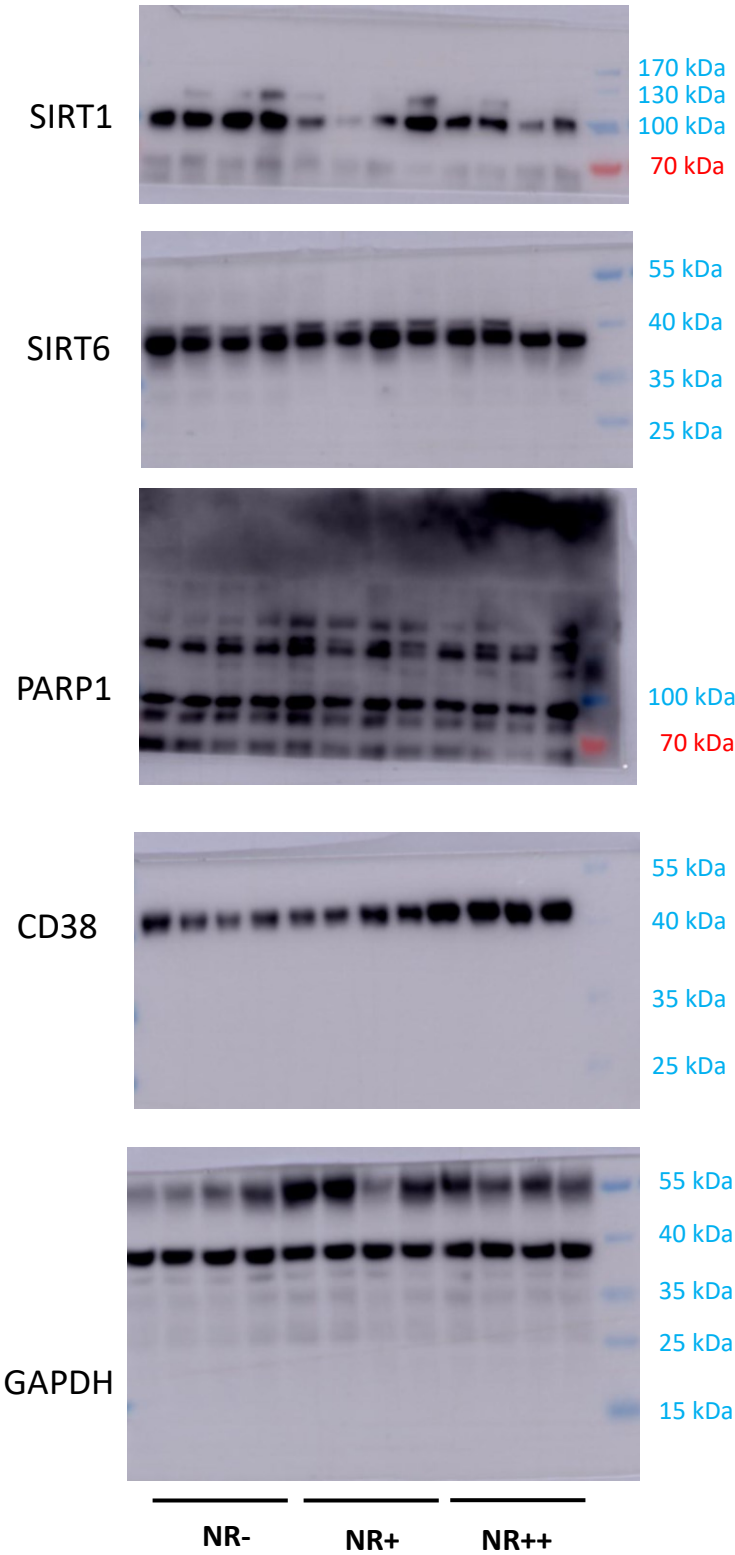

Fig. 4B

PARylation (all gel)

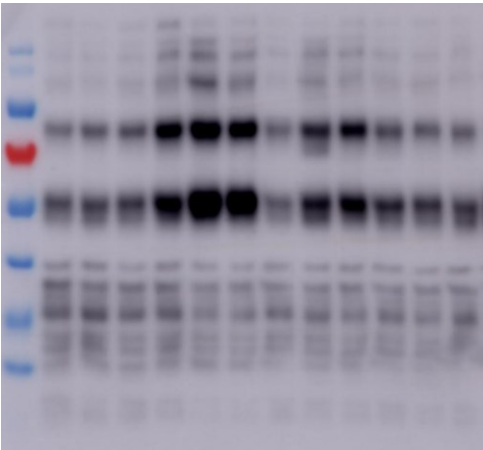

GAPDH (37kDa)

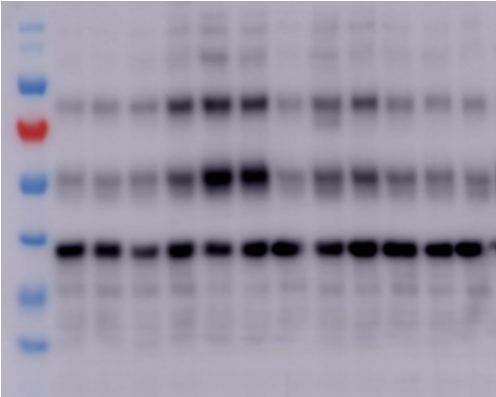

NR- NR+ NR++

Fig. 5C

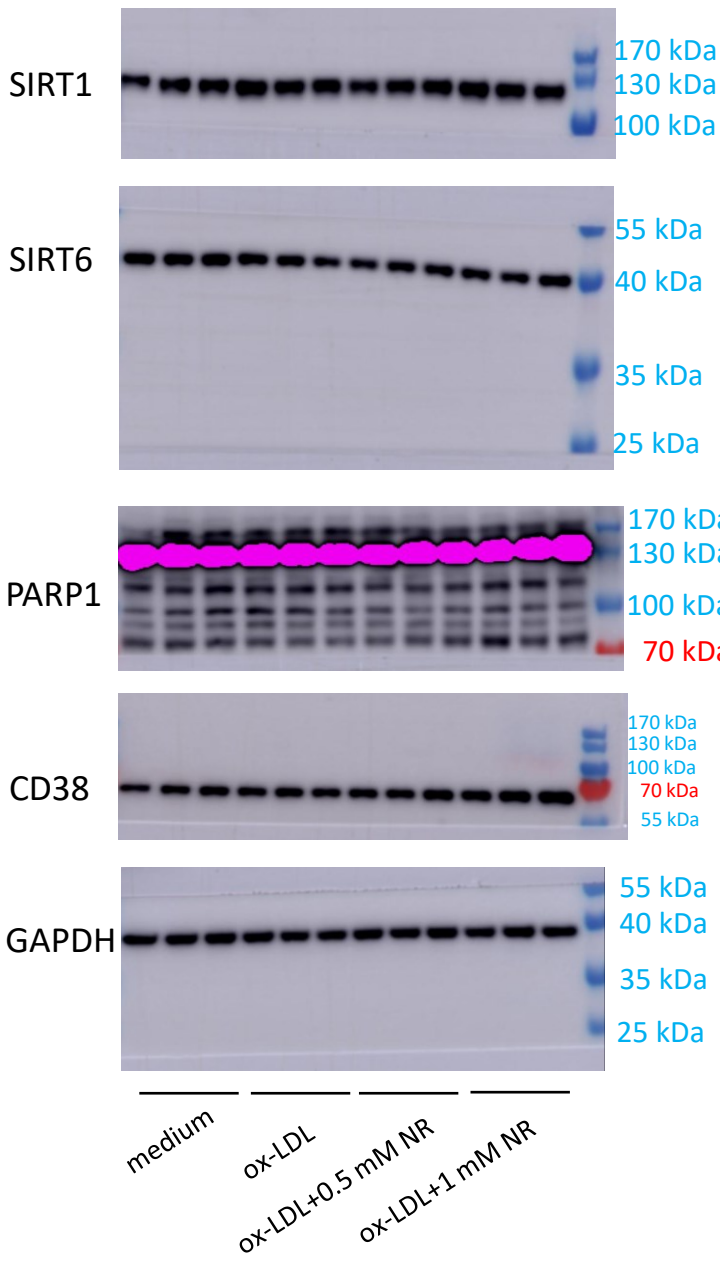

Fig. 5D

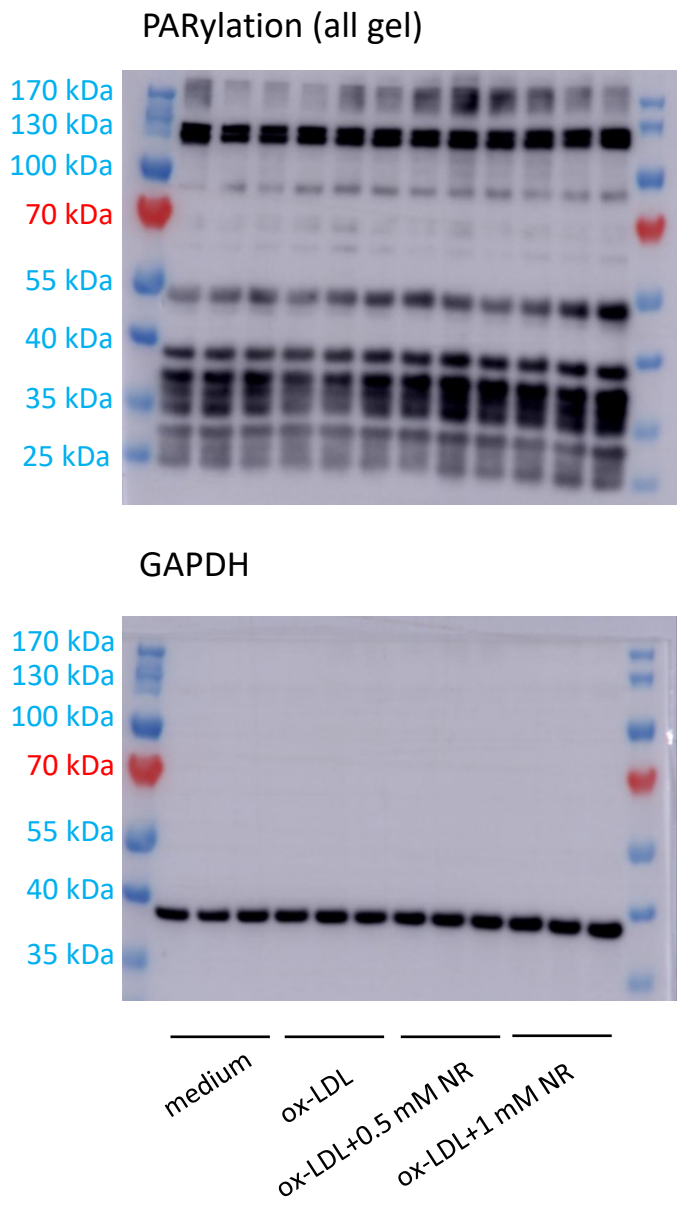

Suppl. Fig. 6C and D

C

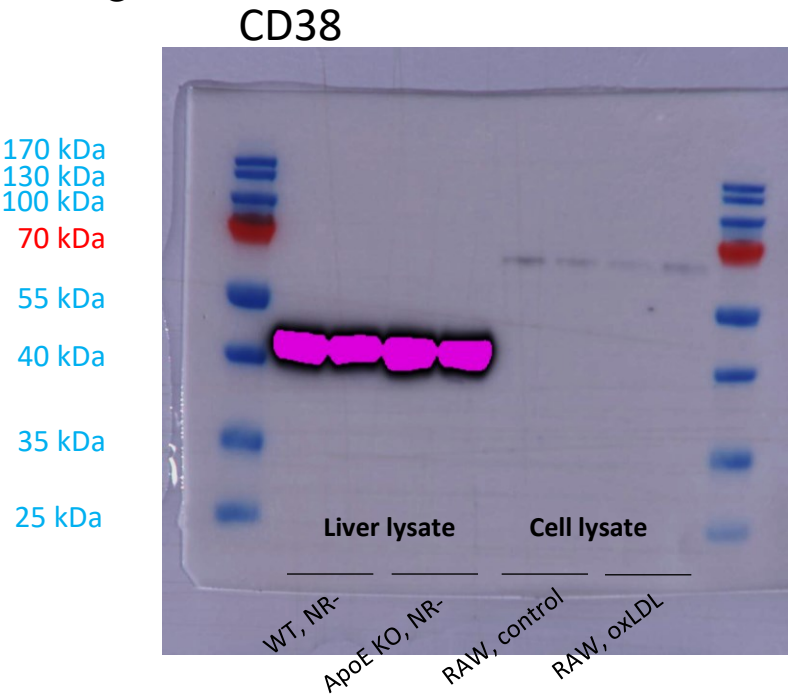

D

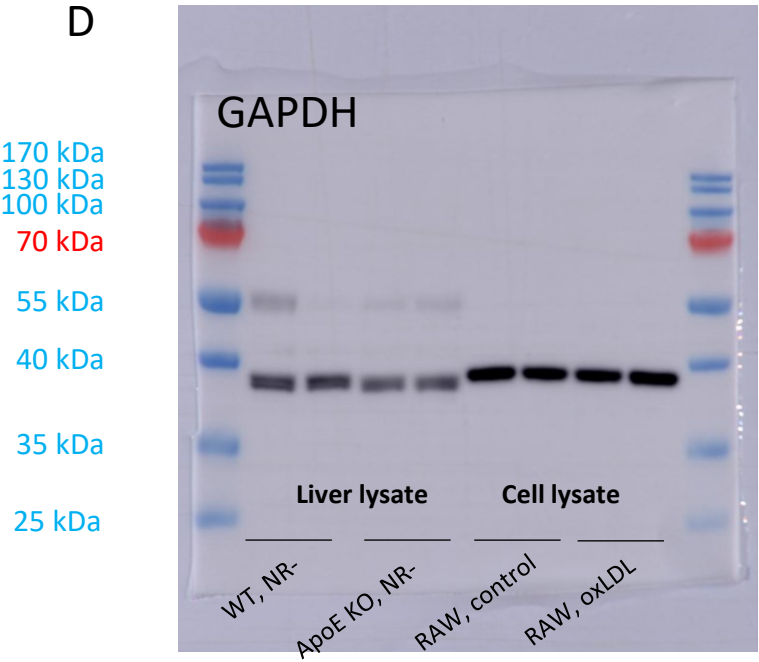

Supplement: Wang et al suppl 4 [file NIHMS2112546-supplement-Wang_et_al_suppl_4.pdf]
